# The Liver-Heart Connection: A Literature Review of Liver Disease as a Risk Factor for Atrial Fibrillation

**DOI:** 10.7759/cureus.38536

**Published:** 2023-05-04

**Authors:** Mukosolu F Obi, Arianna Reinberg Palmar, Vikhyath Namireddy, Frederick N Campos, Hyun Joon Cho

**Affiliations:** 1 Internal Medicine, Wyckoff Heights Medical Center, Brooklyn, USA; 2 Medicine, St. George's University School of Medicine, True Blue, GRD

**Keywords:** metabolic syndrome and endocrinology, nonalcoholic fatty liver disease (nafld), non-alcoholic steatohepatitis (nash), chronic viral hepatitis, hepatic fibrosis, atrial fibrillation (af)

## Abstract

Atrial fibrillation (AFib) is a common type of cardiac arrhythmia, characterized by disorganized atrial electrical activity with features of irregularly irregular heart rhythm and often with rapid ventricular response increasing the risk of stroke and heart failure due to tachyarrhythmia. The pathophysiology mechanism of AFib is either triggered by atrial distension, abnormality in conducting system, catecholamine excess, or increased atrial irritation or automaticity. Risk factors include uncontrolled diabetes, obesity, obstructive sleep apnea, hypothyroidism, and certain stimulants. Based on recent research, liver disease has recently been identified as a risk factor for AFib. Considering the progression of chronic liver disease, this literature review aims to investigate and summarize the relationship between liver disease and AFib and explore clinical interventions that can be utilized to prevent AFib aggravation.

## Introduction and background

An excessive buildup and deposition of extracellular matrix proteins in the liver results in hepatic fibrosis, a degenerative condition that scars the liver and impairs its function. The underlying mechanism of hepatic fibrosis is complex and involves interactions among liver injury, inflammation, immune response, and fibrogenesis [1]. Chronic viral hepatitis, abuse of alcohol, non-alcoholic fatty liver disease (NAFLD), autoimmune hepatitis, metabolic disorders, and exposure to specific medications and chemicals, such as methotrexate, amiodarone, and aflatoxin, are only just a handful of the causes of hepatic fibrosis. Recent studies have demonstrated that extracellular matrix stiffness is also involved in the progression of fibrogenesis. Changes in the extracellular matrix composition and stiffness have been shown to activate signaling pathways that promote the transition of quiescent hepatic stellate cells (HSCs) to myofibroblasts. Myofibroblasts are cells that have contractile properties and secrete collagen, leading to the deposition of fibrotic tissue [1].

The concept of HSC activation is still the cornerstone in understanding the development of hepatic fibrosis. Several studies have emerged with the identification of cellular sources of extracellular matrix, in addition to HSCs, that contribute to the formation of fibrosis [2]. Additionally, it has been discovered that several signaling pathways, including chemokines, adipokines, neuroendocrine, angiogenic, and NADPH oxidase, contribute to the pathophysiology of hepatic fibrosis [2]. There is a role of innate immunity in hepatic fibrosis. The inflammatory response that causes fibrosis is aided by the activation of immune cells, such as neutrophils and Kupffer cells. Additionally, studies have identified the critical role of various transcription factors in regulating the activation of HSCs and myofibroblasts [2]. Hepatic fibrosis may also be brought on by genetic susceptibility, obesity, and certain viral infections such as HIV. Hepatic fibrosis is known to generate oxidative stress, autonomic dysfunction, and systemic inflammation, all of which can result in cardiac remodeling and raise the risk of arrhythmias. While numerous studies have reported a significant association between liver disease and atrial fibrillation (AFib), the underlying mechanisms remain unclear. We analyze several studies that demonstrate a significant correlation, independent of other cardiovascular risk factors. Recognizing this link is crucial for the early diagnosis and treatment of this condition in patients with liver disease. However, more investigation is required to fully understand the underlying mechanisms and to pinpoint possible therapeutic targets for both prevention and treatment.

## Review

To review the literature on the association between hepatic fibrosis and AFib, we performed an electronic search on NIH (with PubMed interface), using the keywords “liver disease,” “hepatic fibrosis,” AND “atrial fibrillation.” A total of 16 studies ranging from 2002 to 2022 were reviewed and synthesized into an overview of how these two conditions may be linked (Table 1).

**Table 1:** Results of the literature review conducted HIV: human immunodeficiency virus, HCV: hepatitis C virus, NASH: nonalcoholic steatohepatitis, NAFLD: non-alcoholic fatty liver disease, MAFLD: metabolic-associated fatty liver disease, AFib: atrial fibrillation, VIP: vasoactive intestinal peptide, HSC: hepatic stellate cell, APD: action potential duration

Authorship and year of publication	Objective	Subtype of liver disease studied	Conclusion
Friedman (2008) [1]	To understand the mechanism behind hepatic fibrosis and its resolution.	HIV/HCV co-infection. HCV. Inflammatory liver disease (NASH),	Understanding, diagnosing, and treating hepatic fibrosis by knowing the fundamental mechanism behind fibrogenesis will help guide treatment and impact prognosis in patients with liver disease.
Friedman (2010) [2]	To understand that other etiologies cause hepatic fibrosis other than HSC activation and knowing these triggers can help develop new therapies for chronic liver disease.	N/A	By understanding the cellular and molecular basis of hepatic fibrosis, targeted therapies involving epigenetic and transcriptional mediators are emerging, etc.
Kang et al. (2020) [3]	To investigate the relationship between AFib and advanced liver fibrosis in patients with ultrasound-screened NAFLD.	NAFLD	As the risk of advanced fibrosis increased, the prevalence of AFib significantly increased.
Wijarnpreecha et al. (2017) [4]	To summarize all available evidence on the possible relationship between NAFLD and AFib.	NAFLD	There is a significantly increased risk of AFib among patients with NAFLD.
Decoin et al. (2022) [5]	To assess the impact of MAFLD and liver fibrosis status on left atrium structure and function.	MAFLD	Liver fibrosis scoring in MAFLD patients is associated with adverse atrial remodeling and AFib recurrences following catheter ablation.
Haghbin et al. (2020) [6]	To review the known pathways and pathophysiology that link AFib and NAFLD.	NAFLD	The relationship between NAFLD and AFib is bidirectional and linked by proinflammatory, pro-oxidant, pro-fibrosing, and atherogenic effects. Additional factors such as dysbiosis of gut flora and anatomical complications of obesity also contribute to the development of both conditions.
Di Stefano et al. (2016) [7]	To present a revision of previous literature about the prevalence, pathophysiological mechanisms, clinical features, and mortality and morbidity of autonomic dysfunction secondary to hepatic cirrhosis.	Cirrhosis	Autonomic dysfunction is common in patients with cirrhosis of the liver and may be associated with increased overall cardiovascular mortality and morbidity. Most cases of autonomic dysfunction resolve after a liver transplant, confirming the central role of portal hypertension and hyperdynamic circulation in the autonomic nervous system.
Liu et al. (2013) [8]	To investigate the role of neurotransmitters in AFib inducibility.	N/A	Neurotransmitters, such as VIP released during vagus nerve stimulation, can promote sustained AFib despite ganglionated plexi ablation and “autonomic blockade,” which may further define the substrate for AFib outside the pulmonary vein-atrial junctions.
Xi et al. (2015) [9]	To investigate the effects of neuronally released VIP on atrial electrophysiologic properties during vagal stimulation.	N/A	VIP contributes to vagal effects on atrial electrophysiologic properties and affects the pathophysiology of vagally induced AFib. Release of VIP in the atria is inhibited by a muscarinic blockade, a novel mechanism by which VIP effects are concealed by atropine during vagal stimulation.
Xi et al. (2013) [10]	To test the hypothesis that VIP alters the atrial electrophysiological properties through its diverse effects on ion channels and thereby affects vulnerability to AFib.	N/A	VIP shortens the atrial APD, increases APD spatial heterogeneity, and slows intra-atrial conduction.
Weber et al. (2013) [11]	To review relevant cellular, subcellular, and molecular mechanisms integral to cardiac fibrosis and consequent remodeling of atria and ventricles with heterogeneity in cardiomyocyte size.	N/A	Myofibroblast activity plays a critical role in the development and progression of cardiac fibrosis, which can ultimately lead to heart failure.
Ho et al. (2014) [12]	To examine the association of Gal-3 and incident AFib in the community.	N/A	Higher circulating Gal-3 concentrations were not associated with an increased risk of developing AFib after accounting for traditional clinical risk factors for AFib development.
Lippi et al. (2015) [13]	To review current evidence on the epidemiological and biological association between galectin-3 and AFib.	N/A	Galectin-3 may be regarded as a promising tool to assist risk stratification and outcome prediction of AFib.
Van et al. (2022) [14]	To investigate the association between liver stiffness, a measure of liver fibrosis, and the risk of developing AFib in the general population.	Fatty liver disease and liver stiffness	Fatty liver disease was not associated with prevalent or incident AFib, while liver stiffness was significantly associated with AFib, especially among those without steatosis.
Liu et al. (2022) [15]	To understand the role of galectin-3 in the progression of liver disease and its contribution to biliary atresia.	Cholestatic liver disease	The elevated level of galectin-3 is associated with an increased risk of progressive liver disease as galectin-3 is involved in profibrotic HSC activation.
Yoeli et al. (2022) [16]	To understand the role of galectin-3 in the progression of liver disease and its contribution to biliary atresia.	Cholestatic liver disease	The elevated level of galectin-3 is associated with an increased risk of progressive liver disease as galectin-3 is involved in profibrotic HSC activation.

Several studies have demonstrated a significant association between liver disease and AFib. A retrospective cross-sectional study investigated the relationship between AFib and advanced liver fibrosis in patients with NAFLD using two non-invasive scoring systems: the NAFLD fibrosis score (NFS) and Fibrosis-4. It screened 6,293 patients with ultrasound for NAFLD and found that 0.9% (59 patients) had AFib. Patients with AFib were older, more likely to be male, had higher BMI and bigger waist circumference, and had a higher prevalence of obesity and diabetes mellitus. Platelet counts, serum albumin, and fasting plasma glucose were significantly higher in the AFib group than in the non-AFib group. The data found that as the risk of advanced liver fibrosis increased, the prevalence of AFib significantly increased [3]. A meta-analysis of five studies (two cross-sectional studies and three cohort studies) with 238,129 participants found an approximately two-fold increased risk of AFib among NAFLD patients compared with subjects without NAFLD, with a pooled risks ratio of 2.06 (95% confidence interval, 1.10-3.85) [4].

A post hoc analysis examined the association between liver fibrosis scores and the incidence of AFib in patients with heart failure with preserved ejection fraction (HFpEF). The findings suggest that advanced liver fibrosis is associated with increased new-onset AFib incidence and may be a novel predictor of new-onset AFib in patients with HFpEF. Particularly, it was the elevated NFS score that was significantly associated with the incidence of AFib and had moderate predictive ability in predicting such occurrence among patients with HFpEF. The event of an AFib was linked to patients who had a larger mitral regurgitation (MR) jet area, higher left atrial area, higher MR jet area‐to‐left atrial area ratio, lower peak A wave velocity, and lower left atrial volume index. Additionally, diabetes, age, alanine transaminase, and aspartate aminotransferase were positively associated with AFib incidence, while platelets were inversely related. The authors suggest that liver fibrosis may be a new component in risk scores for AFib in HFpEF, and clinicians should consider incorporating liver fibrosis score assessment into their AFib risk scoring models to prevent new-onset AFib and monitor HFpEF progression. Additionally, physicians should be careful when administering drugs like statins, amiodarone, and warfarin since they may influence liver function and may cause changes in transaminase and platelet levels, which could suggest changes in liver fibrosis [15]. Likewise, another study examined the association between liver fibrosis scores in patients with metabolic-associated fatty liver disease (MAFLD) and the incidence of adverse atrial remodeling and AFib recurrence after catheter ablation. The findings indicate that higher liver fibrosis scores in MAFLD patients are linked to adverse atrial remodeling and a greater risk of AFib recurrence following catheter ablation. The study proposes that liver fibrosis score assessments could be useful in identifying patients at a higher risk of AFib recurrence and could aid in determining personalized treatment strategies for MAFLD patients undergoing catheter ablation. Furthermore, the findings revealed that, as determined by NFS throughout the follow-up period, patients with "MAFLD w/severe fibrosis" had a considerably greater rate of AFib recurrence (77%) compared to those with ambiguous fibrosis (32.5%) or without hepatic fibrosis (17.5%). Remarkably, the authors found that atrial fibrosis, a chief component for the development of AFib, was especially increased in patients presenting MAFLD with a high liver fibrosis score [5].

Putative mechanism

Systemic inflammation, autonomic dysfunction, oxidative stress, and venous congestion may underlie the association between liver disease and AFib. According to a study on the potential processes relating NAFLD and AFib, obesity, insulin resistance, inflammation, oxidative stress, autonomic dysfunction, and changes in gut microbiota may be common risk factors causing both diseases. As a result, lifestyle changes like losing weight and exercising, as well as drugs that target inflammation and insulin resistance, may have therapeutic potential for both NAFLD and AFib [5]. One mechanism linking AFib and NAFLD in obese individuals is the inflammatory and oxidative system. The innate immune system produces reactive oxidation species through myeloperoxidase enzymes, which are closely linked to inflammation and oxidative stress. In obese individuals, there is decreased release of anti-inflammatory adiponectin and an increased release of pro-inflammatory mediators such as interleukin 6 (IL-6), tumor necrosis factor-alpha (TNF-α), and C-reactive protein (CRP) from adipose tissue. This leads to a pro-inflammatory environment, resulting in cell death, fibrosis, and the progression of both AFib and NAFLD. These all contribute to cardiac remodeling, with structural and cellular changes that may propagate AFib.

Furthermore, individuals with NAFLD have active renin-angiotensin systems (RAS), which can lead to hypertension and cardiac fibrosis. Angiotensin II, a pro-inflammatory component of RAS, causes an increase in cytokines and worsens NAFLD through hepatic infiltration of inflammatory cells. Angiotensin II also promotes oxidative stress and leads to insulin resistance and cardiac fibrosis mediated by transforming growth factor beta (TGF-β), resulting in left atrial electrical and mechanical remodeling [6].

Atrial and liver fibrosis share similar mechanisms that lead to the activation of fibroblasts within the myocardium or HSCs in the liver; these are responsible for collagen formation. Macrophages are believed to contribute to fibroblast activation through TGF-β. Specific to the MAFLD study, this condition can be seen as a form of systemic low-grade inflammation; thus, the development of fibrosis in the heart could be a result of prior immune cell activation. Adipose tissue, particularly epicardial adipose tissue, has been suggested as a major factor in atrial remodeling and AFib incidence. Adipokines, such as adiponectin, have been found to have an inverse correlation with indexed left ventricular mass and a positive correlation with indexed left atrial volume and E/e′ ratio, a parameter used in echocardiography to assess the left ventricular filling pressure. Furthermore, elevated leptin levels and decreased adiponectin levels are seen in individuals with MAFLD and liver fibrosis. This suggests that atrial remodeling caused by adipose tissue may also be a factor in MAFLD. These patients exhibit a higher risk for AFib recurrence following catheter ablation, indicating the importance of the management of MAFLD on left atrium remodeling and AFib ablation outcomes [5].

Autonomic dysfunction is another potential mechanism linking liver disease and AFib. Autonomic dysfunction in cirrhotic portal hypertension may result from hyperdynamic circulation. Contributing factors include regenerative fibrotic nodules that release endothelin and thromboxane A2, leading to vasoconstriction within hepatic sinusoids and reduced response to vasodilator stimuli. In the process, the sympathetic nervous system and RAS are subsequently activated, increasing heart rate, myocardial contractility, left ventricular ejection fraction, and cardiac output. This results in an increase in the synthesis of vasodilators. Liver dysfunction and portal-systemic shunts increase vasodilators, reducing peripheral vascular responsiveness to vasoconstrictors. Vagal dysfunction is caused by increased angiotensin II, impaired sodium urinary excretion, and intravascular fluid retention, maintaining hyperdynamic circulation. All these result in decreased regulation of heart rate and elevated sympathetic tone, which lead to oxidative stress and the endothelium and myocardial damage seen in cirrhotic cardiomyopathy. Of note, most cases of autonomic dysfunction resolved after liver transplantation, confirming the central role of portal hypertension and hyperdynamic circulation in the autonomic nervous system [7]. Studies have indicated that a decrease in liver function can cause bile acid, ammonia, and cytokines to build up in the bloodstream and negatively impact the neurological system. Increased amounts of neuropeptides have also been linked to advanced liver fibrosis, which may aid in the onset of AFib. For instance, a vasoactive intestinal peptide is hypothesized to be the cause of vagal-mediated AFib [8-10].

Galectin-3 is a beta-galactoside-binding protein that is involved in several biological processes, including cell adhesion, migration, proliferation, apoptosis, and immunological response. The liver and the heart are two tissues where it is expressed among others in minimal amounts. Galectin-3 production has been observed to be elevated or increased in inflammatory and fibrotic processes, as seen in liver disease [13,16]. Galectin-3 is upregulated in cirrhotic livers, especially in the regenerative nodules. The main regulator of liver fibrosis is HSCs. HSCs phagocytose apoptotic bodies in response to liver damage, differentiating into an active, myofibroblast phenotype that produces transforming growth factor-1 (TGF-1) and releases collagen into the extracellular matrix within the liver, resulting in the formation of fibrotic ridges and bridging fibrosis that are indicative of advanced liver fibrosis and cirrhosis [1,16]. The activation of profibrotic HSCs depends on galectin-3 based on an experimental study; in response to the treatment of carbon tetrachloride, HSCs from galectin-3 mutant mice showed reduced phagocytosis, TGF-1 synthesis, and procollagen production. Recombinant galectin-3 can counteract this impact in an in vitro experiment, proving that extracellular galectin-3 is essential for HSC activation [16].

Another study found that higher circulating galectin-3 concentrations were associated with an increased risk of developing AFib over a 10-year period in age- and sex-adjusted prospective analysis. In the study conducted, elevated serum galectin-3 was found to be a predictor of left atrial fibrosis, reduced left atrial volume, and decreased left ventricular ejection fraction. Upregulation of galectin-3 leads to the activation of HSC. Activated macrophages also release galectin-3 which also promotes fibroblast activation and proliferation in cardiac tissue leading to myocyte remodeling, and dysfunction leading to AFib [12]. Thus, the study concluded that the level of galectin-3 can be used for risk stratification and outcome in patients with AFib.

Oxidative stress can stimulate fibrosis in the atria, which can disrupt electrical signaling by creating areas of slow conduction, unidirectional block, and reentry. Additionally, oxidative stress can impair the ability of proteins and enzymes involved in cardiac electrical signaling and ion transport, resulting in irregular electrical activity and arrhythmia. To prevent and cure AFib in this population, the authors propose that lowering oxidative stress in patients with liver illness may be a suitable therapeutic target.

However, higher liver stiffness, especially in individuals without steatosis, was associated with prevalent AFib. This association could be due to venous congestion instead of fibrogenesis, but more research is needed to confirm this. The study suggests that liver stiffness may be increased due to factors not related to liver disease, and it is necessary to investigate whether the same liver stiffness cut-offs for fibrosis are suitable for participants with AFib. Therefore, individuals with high liver stiffness, even without overt liver disease, should be evaluated for their cardiovascular health [14].

## Conclusions

Numerous studies have shown that there is a significant correlation between liver disease and AFib. The relation is complex and multifactorial, mediated by various factors such as inflammation, oxidative stress, and structural remodeling of the atria all in response to progressive liver disease. Elevation in galectin-3 as seen in worsening liver disease was found to be pro-fibrotic and pro-inflammatory predisposing to increase the risk of AFib. The suggestion is that measuring the level of galectin-3 can be a predictive value to monitor for the possible onset of new AFib in patients with advanced liver disease. Understanding the epigenetic and transcriptional mediators involved in the activation of HSC can help with the development of antifibrotic drugs or therapies, as treating liver disease can help decrease the risk of developing AFib. Further research is needed to better devise a potential therapeutic target for the prevention and treatment of AFib in patients with liver disease. A possible avenue of investigation is finding noninvasive markers of fibrogenic activity that can help monitor identify and monitor disease progression and eventually help accelerate the development of antifibrogenic therapies that can help decrease liver disease progression increasing the risk of AFib. This study affirms the consideration of screening for AFib in patients with progressive liver cirrhosis especially when they present with decompensated liver cirrhosis. Clinicians should be aware of the link between liver disease and AFib as identifying patients on time may benefit patients from early intervention to prevent and treat AFib.
